# Construction of an immune-related lncRNA pairs model to predict prognosis and immune landscape of lung adenocarcinoma patients

**DOI:** 10.1080/21655979.2021.1953215

**Published:** 2021-07-21

**Authors:** Junhui Liu, Hao Wu, Zhaojia Gao, Ming Lou, Kai Yuan

**Affiliations:** a Division of Thoracic Surgery, The Affiliated Changzhou No.2 People’s Hospital of Nanjing Medical University, Changzhou, China; b School of Medicine, Dalian Medical University, Dalian, China; c Heart and Lung Disease Laboratory, The Affiliated Changzhou No.2 People’s Hospital of Nanjing Medical University, Changzhou, China

**Keywords:** TCGA, immune-related lncRNA, lung adenocarcinoma, immune cell infiltration, ICI-related biomarkers, chemotherapeutics

## Abstract

The model of immune-related lncRNA pairs (IRLPs) seems to be an available predictor in lung adenocarcinoma (LUAD) patients. The aim of our study was to construct a model with IRLPs to predict the survival status and immune landscape of LUAD patients. Based on TCGA-LUAD dataset, a risk assessment model with IRLPs was established. Then, ROC curves were used to assess the predictive accuracy and effectiveness of our model. Next, we identified the difference of survival, immune cell infiltration, immune checkpoint inhibitor-related (ICI-related) biomarkers, and chemotherapeutics between high-risk group and low-risk group. Finally, A nomogram was built for predicting the survival rates of LUAD patients. 464 LUAD samples were randomly and equally divided into a training set and a test set. Six IRLPs were screened out to construct a risk model. K-M analysis and risk-plot suggested the prognosis of high-risk group was worse than low-risk group (p < 0.001). Multivariate analysis shows risk score was independent risk factor of LUAD (p < 0.001). In addition, the expression of immune cell infiltration, ICI-related biomarkers, chemotherapeutics all demonstrate significant difference in two groups. A nomogram was built that could predict the 1-, 3-, and 5-year survival rates of LUAD patients. Our immune-related lncRNA pairs risk model is expected to be a reliable model for predicting the prognosis and immune landscape of LUAD patients.

## Graphical Abstract

**Figure:**
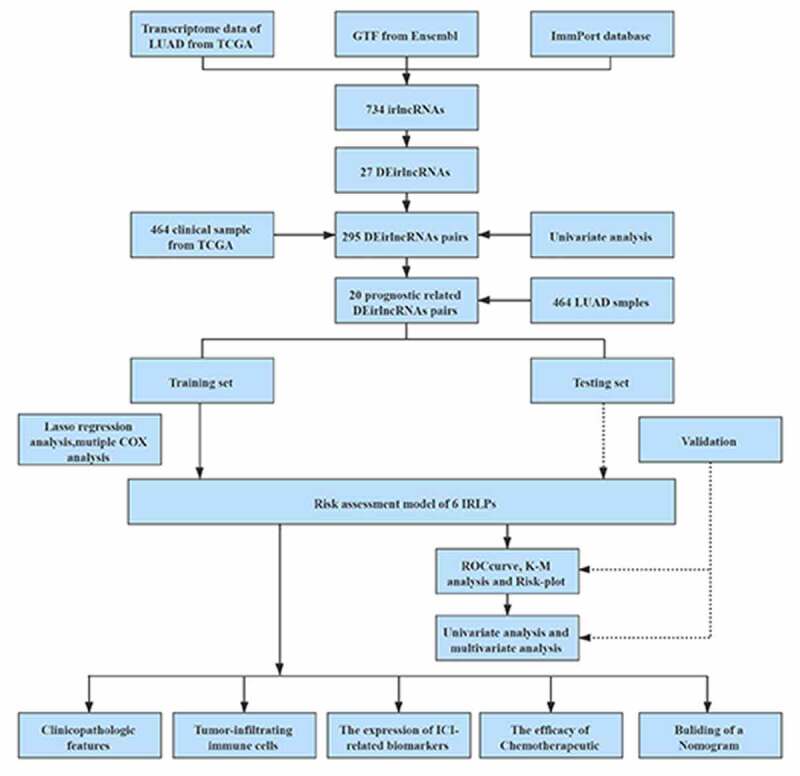

## Introduction

Lung cancer is the most common type of tumor-related diseases that causes death [1]. In addition, lung adenocarcinoma (LUAD), which accounts for approximately 40% of lung cancer, has long been come into focus worldwide [2]. Over the past decade, although treatment of LUAD has been increasing and the clinical outcome has been improved, the 5-year survival rate of LUAD patients is still low with only 18%, which may put down to that lung adenocarcinoma tends to tumor metastasis at an early stage. Therefore, it is urgent for us to explore a better way to evaluate the prognosis of LUAD patients.

LncRNAs, long noncoding RNAs, which account for approximately 80% of the human transcriptome are more than 200 nucleotides in length [3]. LncRNAs perform a wide range of biological functions, such as chromatin interactions, cell differentiation, transcriptional regulation, and RNA processing [4]. In addition, increasing evidence shows that lncRNAs cannot only play vital roles in tumorigenesis and metastasis, but also involve in some immune activities, such as immune activation, immune escape, and immune cell infiltration [5].

Study shows that Long noncoding epidermal growth factor receptor (lnc-EGFR) can promote hepatocellular carcinoma growth by inducing Treg differentiation, which offering a potential therapeutic target for hepatocellular carcinoma [6]. Chen et al. found that with the help of C-C Motif Chemokine Ligand 2(CCL2) and vascular endothelial growth factor C (VEGF-C) excretion, Lymph Node Metastasis Associated Transcript 1 (LNMAT1, a kind of lncRNA) can recruit macrophages into the tumor, then promotes lymphatic metastasis in bladder cancer [7]. On the basis of that, more and more researches demonstrate that lncRNAs were related to immune-related genes. Researchers try to construct the model about this aspect and further analysis the function of the model in tumors. For instance, Jiang et al. screened out three immune-related lncRNAs (irlncRNAs, IRLs) to construct an immune-related risk score model and demonstrated the prognostic value of this model in renal cell carcinoma [8]. Further, Li et al. constructed two irlncRNA clusters and indicated the significant difference of immunosuppressive tumor microenvironment (TME) and mutation frequency between two clusters, which would be conducive to understanding immune molecular mechanisms of LUAD. They also made a comparison on the expression of different immune cells (Macrophages M0, Macrophages M2, Mast cells activated, Neutrophils) between two clusters, and all the results presented the significant difference [9]. Additionally, Xu et al. establish a prognostic model with three immune-related genes in stage I–II LUAD patients, and indicate the model has a significant correlation with some biomarkers of immune checkpoint inhibitors (ICIs) [10].

Nowadays, the research of immunosuppressive therapy is a hot topic, and several ICIs have already been used in clinical practice and have played an effective role in some tumors. Ipilimumab, a kind of ICI that target the cytotoxic T-lymphocyte antigen-4 (CTLA-4) became the first immunocheckpoint inhibitor approved for the treatment of inoperable or advanced melanoma [11]. Nivolumab and Pembrolizumab, which can target programmed cell death 1 (PD-1, PDCD1), and Durvalumab, anti-programmed cell death-ligand 1 (CD274, PD-L1) inhibitors that are all approved for the treatment of some NSCLC patients with disease progression after targeted therapy [12]. These immunocheckpoint inhibitors was also used in other tumors, such as hepatocellular carcinoma [13] and esophagus cancer [14].

Research shows that it is more accurate to predict the prognosis of cancer patients through combing two biomarkers together than only simple genes [15]. Compare to the model constructed by Liu et al in LUAD [16], we construct a novel immune-related lncRNA model with the way of ‘pairing’. The present study aims construct a reliable risk level model based on immune-related lncRNA pairs for the prognosis of LUAD, explore the role of our model in LUAD immune infiltration and clinical pharmacotherapy, develop a viable nomogram to predict prognosis of LUAD.

## Materials and Methods

### Data Source and Processing

Transcriptome profiling (RNA-seq) FPKM (reads per kilobase per million) data (including 535 tumor sample and 59 normal sample) and clinical data of LUAD were downloaded from The Cancer Genome Atlas (TCGA, https://tcgadata.nci.nih.gov/tcga/;LUAD), then were processed by the Perl software (v5.32.1.1, https://www.perl.org/). For distinguishing the mRNAs and lncRNAs, GTF files were obtained from Ensembl (http://asia.ensembl.org). The ImmPort database (http://www.immport.org) was used to retrieve immune-related genes (ir-genes) list. Next, the ir-genes and lncRNAs were merged, then immune-related lncRNAs (irlncRNAs, IRLs) were filtered out by the R software (v4.0.3, https://www.r-project.org/). After that, we performed a differential coexpression analysis to classify differentially expressed irlncRNAs (DEirlncRNAs) (FDR <0.01, logFC >2). We constructed gene-pairs with DEirlncRNAs. Then these immune-related lncRNAs pairs (IRLPs) were used to construct a 0-or-1 matrix by the following methods: the IRLPs were scored as ‘1’ if the expressions were IRL1> IRL2, otherwise, they would ‘0.’ If the number of IRLPs of which expression quantity was 0 or 1 come up to 20% of total pairs, it was considered a valid match.

### Development of a Prognostic Risk Assessment Model

IRLPs and clinical data were combined, then the univariate Cox regression analysis was presented for selecting out prognostic-related IRLPs. Then, the 464 samples were classified into training set and test set by the ‘caret’ package of R software in a 5:5 ratio, A Lasso regression method was applied to filter optimal candidates and multivariate Cox regression analysis were performed to establish the Cox risk assessment model by R packages (‘survminer,’ ‘glmnet’) in the training set. The risk score model for patients was established as follows: IRLP 1× Expression IRLP 1+ IRLP 2× Expression IRLP 2+ … + IRLP n× Expression IRLP n. (IRLP n was the correlation coefficient of the Cox regression risk model for the target IRLP, Expression IRLP n was the expression value of each optimal prognostic IRLP). Finally, the samples were classified into high-risk group and low-risk group respectively according to the median risk score in the two sets.

### Validation of the Risk Model in the Testing Set

First, in the training set, the ROC curve was plotted and the AUC value of our model was calculated for validating the sensitivity and specificity of the model by the R packages (‘survival ROC’). K-M survival curve and risk curve was plotted for analyzing the prognostic correlation of high- or low-risk group. The ‘survival’ R package, ‘survminer’ R package were used in these operations. Additionally, the univariate and multivariate Cox regression analyses were performed to confirm the clinical validation of the constructed risk model. The R packages ‘ComplexHeatmap,’ ‘limma,’ and ‘ggupbr’ were used in these steps. The ROC curve of different clinical characteristics was plotted, and ‘survival ROC’ R package was used. The procedures above were all performed in the testing set for validating our model.

### Clinical Correlation Analysis of the Model

For calculating the association between our risk model and clinicopathological characteristics, the *X^2^* test was performed and the band diagram was plotted, which was marked as follows: <0.001 = ***, <0.01 = ** *, and <0.05 = *. Wilcoxon signed-rank test was also used to analyze the risk score difference in different clinicopathological characteristics.

### Exploration of Tumor-Infiltrating Immune Cells and ICI-related biomarkers

The expression of tumor-infiltrating immune cells associated with LUAD from TCGA were obtained through the online website (http://timer.cistrome.org). Several currently acknowledged methods such as XCELL, TIMER, QUANTISEQ, MCPCOUNTER, EPIC, CIBERSORT−ABS, CIBERSORT in this website were used to explore the relationship between the risk score and the immune infiltration statues. The bubble chat and the box chat were plotted. In the procedure, the R packages such as ggplot2 and ggtext were used. In addition, the expression situation of ICI-related biomarkers in the LUAD were presented with vioplot by the R ggpubr package.

### Excavation of the Function of Risk Model to the Chemotherapeutics

Several commonly used chemotherapeutic drugs such as Docetaxel, Gefitinib, and Paclitaxel are recommended for LUAD treatment. The sensitivity difference of high- or low-risk group to these drugs were analyzed by Wilcoxon signed-rank test. The R packages pRRophetic and ggplot2 were used.

### Building and Validation of a Nomogram

A nomogram was built for survival prognosis and A calibration curve plot was used to validate the predictive accuracy and the concordance index (C-index). The ‘rms’ package was used here.

## Results

The present study aims to identify the correlation between a risk assessment model based on immune-related lncRNA pairs and prognosis of LUAD patients, and explore the potential role of the model in immune cells infiltration and clinical therapy. First, six prognosis-related immune lncRNA pairs were screened out to construct a risk assessment model. Then, ROC curve, K-M survival curve, and risk status curve were plotted. The model was validated by internal cohort. Next, the impact of the model in clinicopathologic features were analyzed. Further, the correlation between immune infiltration, Immunosuppressive Molecules, and Chemotherapeutics were investigated. Finally, a prognostic nomogram was developed and calibrated.

### Data Acquisition and Establishment of a Risk Assessment Model

**Figure 1.**
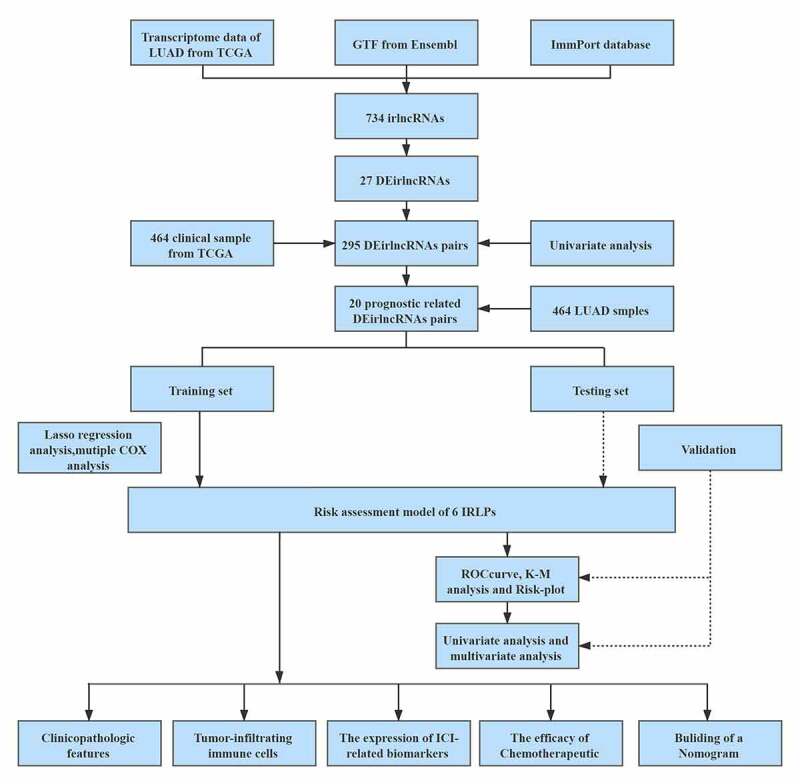
The framework of this study

**Figure 2.**
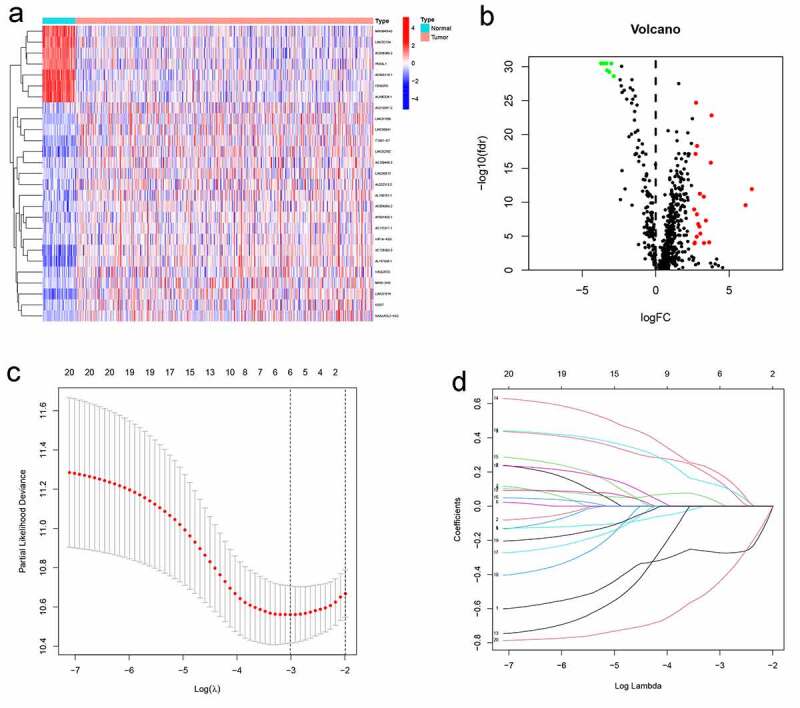
The differentially expressed immune-related lncRNAs (DEirlncRNAs) gained, and LASSO analysis used. (a, b) The 27 DEirlncRNAs were shown in heatmap and volcano plot, respectively. The green, red, and black dots mean downregulated and upregulated genes and no differential expression, respectively. (c, d) Cross-validation for the selection of optimal parameter (lambda) and LASSO coefficient profiles of the candidate DEirlncRNAs pairs, respectively

Figure 1 presents the flow diagram of this study. First, the transcriptome profiling data (including 535 tumor and 59 normal sample) and clinical data of LUAD (include 464 samples) were downloaded from The Cancer Genome Atlas (TCGA) database. Then, the data were annotated and 734 irlncRNAs were identified by the coexpression analysis between known ir-genes and lncRNAs. 27 differentially expressed irlncRNAs pairs were screened out among them (Figure 2(a-b)). Next, according to the iteration loop and a 0-or-1 matrix screening, 295 valid differentially expressed IRLPs were identified (Table S1). We combined these IRLPs with the clinical data then 20 prognosis-related IRLPs were retrieved (Table S2). Next, we divided the 464 LUAD samples into training set and test set. In the training set, a modified Lasso regression analysis and multiple regression analysis were performed (Figure 2(c-d)). 6 DEirlncRNAs pairs were extracted to construct a Cox proportional hazards model (Table 1).

**Table 1.** Six IRLPs and coefficients in the prognostic risk assessment model

Gene pairs	Coef	Gene pairs	Coef
AL590226.1|ITGB1-DT	−0.272620431506035	ITGB1-T|AC131971.1	0.143614111251972
ITGB1-DT|AL157838.1	0.0193198554782944	LINC00941|LINC02362	0.189921097107668
AC026369.3|HIF1A-AS3	−0.444557438396472	ITGB1-DT|LINC00513	0.119044206656088

IRLPs: immune-related lncRNA pairs

### Validation of the Risk Assessment Model

**Figure 3.**
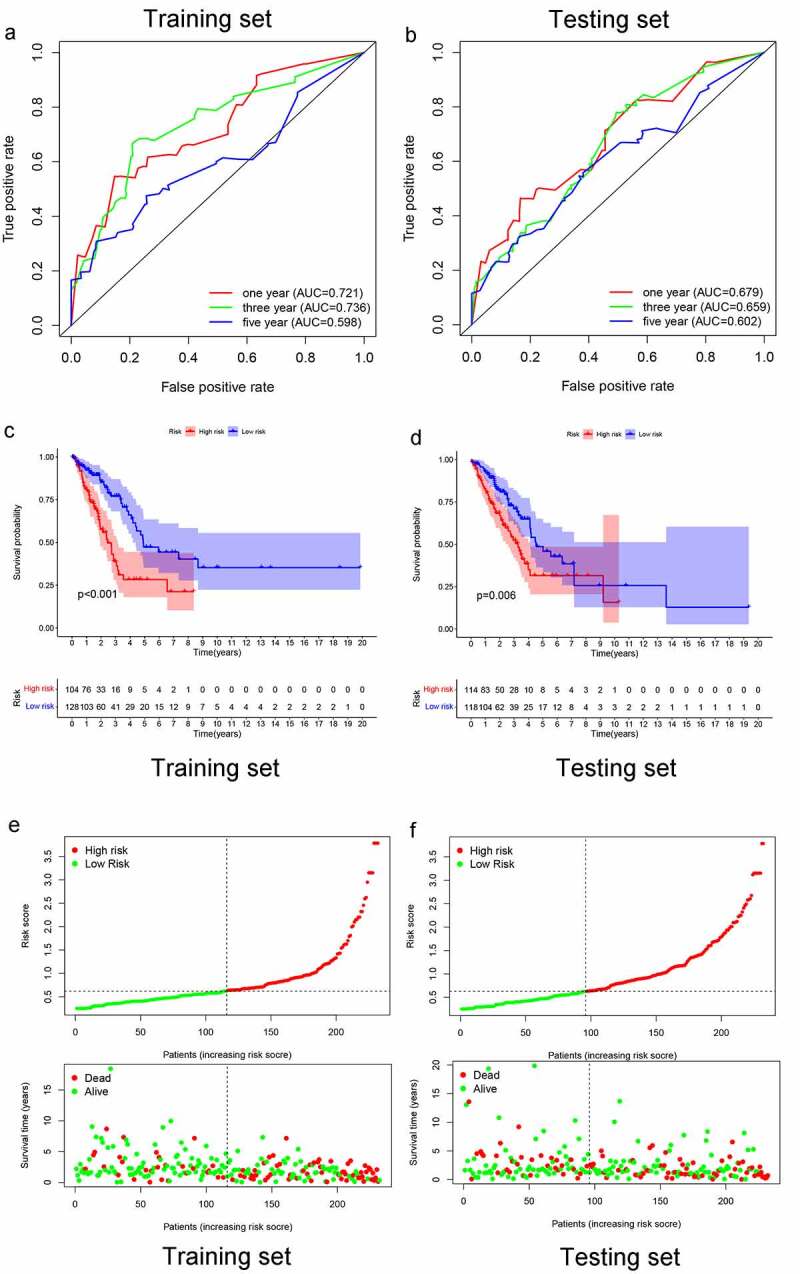
1-, 3-, and 5-year ROC curves, K-M survival curves, and risk-plots were presented in the training set and testing set. (a, b) ROC curves show that the AUC value of 1- and 3-year were better than 5-year in the training set and testing set. (c, d) K-M survival analysis in the training set and testing set. (e, f) Risk scores and survival outcome of each case are shown in the training set as well as testing set

**Figure 4.**
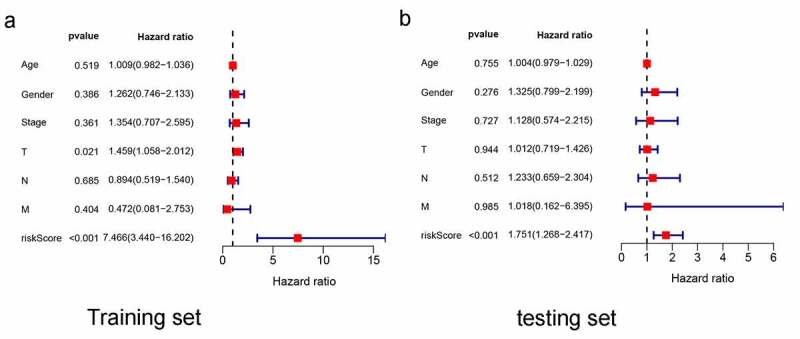
Multivariate independent prognosis analyses of training set and testing set. (a) T stage and risk score were independent predictor in the training set. (b) only risk score was independent predictor in the testing set

In the training set, AUCs for 1-, 3-, and 5-year OSs were 0.7216, 0.736, and 0.598, respectively (Figure (3a)), indicating that the risk signature could predict the 1-, 3-year survival rates for the LUAD patients better than the 5-year OS rates. Similar results were found in the testing set, of which the AUCs for 1-, 3-, and 5-year OSs were 0.679, 0.659, and 0.602, respectively (Figure 3(b)). Next, K-M survival analysis was performed, the survival time of patients in the high-risk group was significantly shorter than that in the low-risk group (p < 0.001 in the training set, and p = 0.006 in the test set) (Figure 3(c-d)). As the risk score increased, the death count increased, which suggest that the clinical outcome of high-risk group was inferior to that of low-risk group either in training set or testing set (Figure 3(e-f)). Further, univariate and multivariate prognostic analysis were performed in the training set, and the result of multivariate independent prognosis analyses shows that T stage (p = 0.021) and risk score (p < 0.001) were predicted as independent prognostic factors (Figure 4(a)), while only risk score (p < 0.001) showed a significant correlation with prognosis in the test set (Figure 4(b)).

### Clinical Analysis between the Risk Assessment Model and the Other Clinical Variables

**Figure 5.**
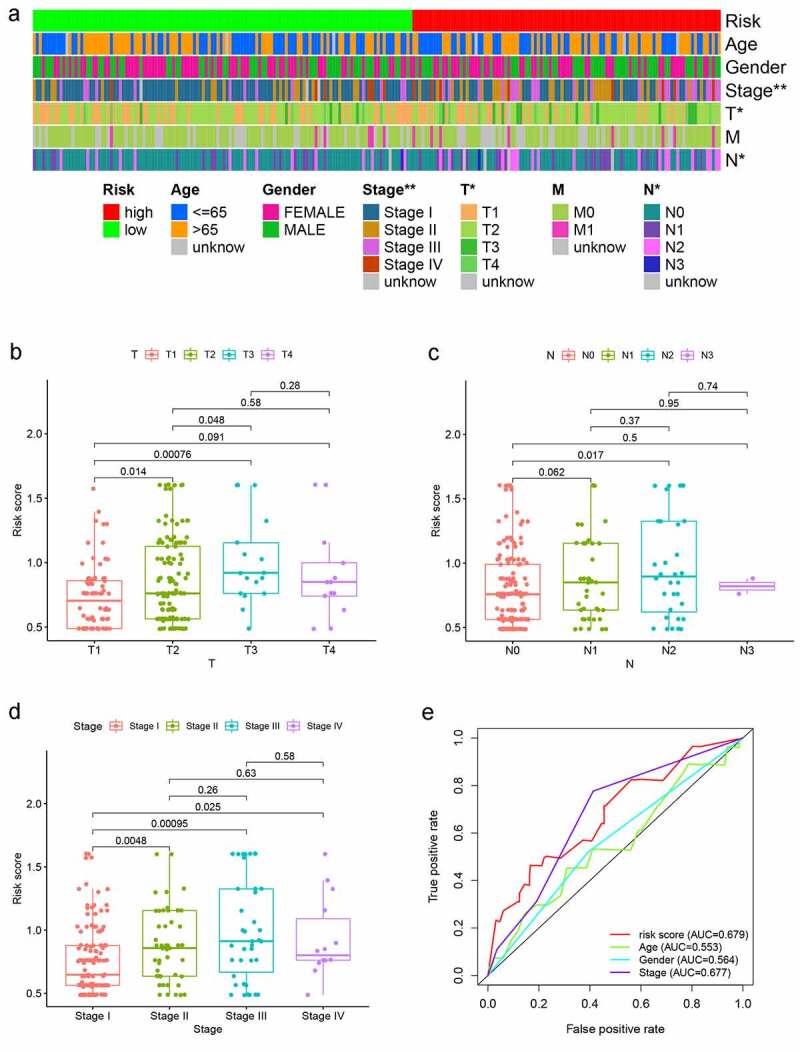
Analysis between clinicopathological features and the risk model. (a–d) A strip chart along with the scatter diagram showed that T stage and N stage, and clinical stage were significantly associated with the risk score. (e) Risk score curve has the maximum AUC value (AUC = 0.679) compared to the other clinicopathological features

In the training set, the relationship between clinicopathological features and risk score was analyzed by *X*^2^ text and the T stage, clinical Stage showed the significant difference in two different risk groups (Figure 5(a). Further, the T stage, N stage, and clinical Stage were presented significantly correlation to the risk score by Wilcoxon signed-rank test (Figure 5(b-d)). The ROC curves of different clinical characteristics and risk score were plotted. It shows that our risk score curve has the maximum AUC value (AUC = 0.679), which suggests that our model is superior to other clinical traits in predicting survival of LUAD patient (Figure 5(e)).

### Evaluation of Tumor-Infiltrating Immune Cells and Immunosuppressive Molecules by the Risk Model

**Figure 6.**
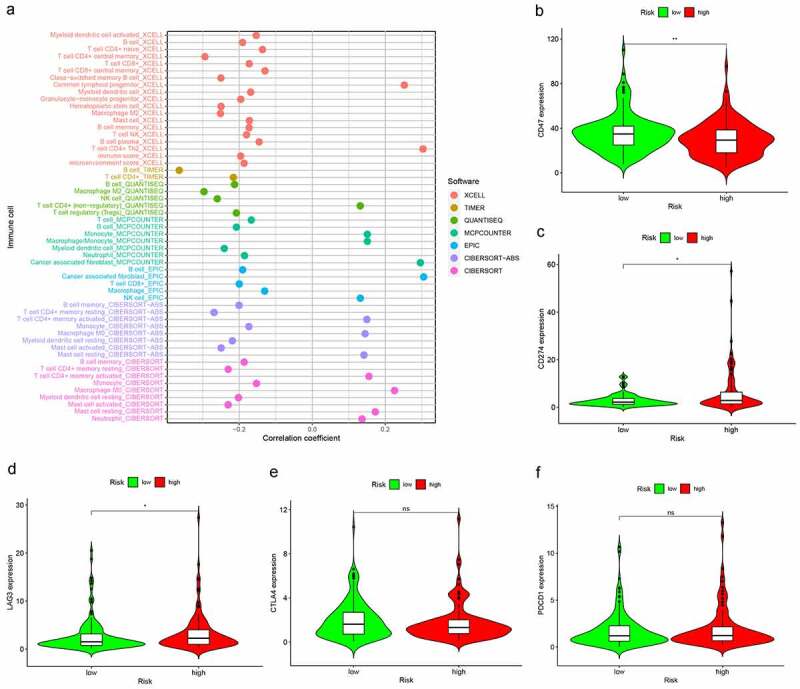
Risk model in tumor-infiltrating immune cells, and ICI-related biomarkers. (a) The high-risk group was more positively correlated with macrophages M0, Neutrophil, T cell CD4+ memory activated as shown in bubble chart. (b–f) The expression of CD47, CD274, and LAG3 were statistically different between high- and low-risk groups, while not in CTLA4, PDCD1 (p < 0.001 = ***, <0.01 = **, and <0.05 = *)

As shown in Figure 6a, the high-risk group was more positively correlated with macrophages M0, Neutrophil, T cell CD4+ memory activated, while negatively correlated with B cell, T cell CD8+ et al. In addition, ICIs are administered for treating LUAD in clinical practice, therefore, we excavated the correlation between ICI-related biomarkers and risk model, and the expression difference only found in integrin-associated protein (CD47, IAP) (p < 0.01), CD274 (p < 0.05), Lymphocyte-activation gene 3 (LAG3) (p < 0.05) while not in CTLA4, PDCD1 (Figure 6(b-f)).

### Estimation of the Function of Risk Model to Chemotherapeutics

**Figure 7.**
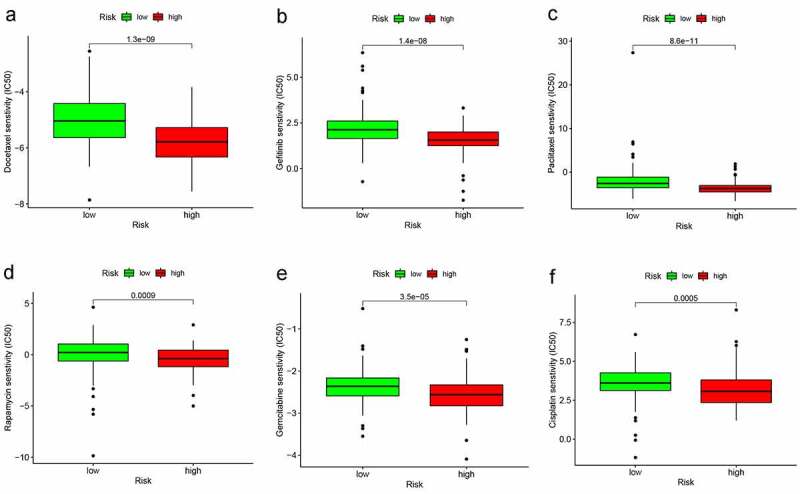
The different sensitivity of high- and low-risk groups to chemotherapeutics drugs. (a–d) The low-risk group was associated with a higher half inhibitory concentration (IC_50_) in Docetaxel, Gefitinib, Paclitaxel, Rapamycin, Gemcitabine, and Cisplatin

In order to estimate the function of our model in the clinical, we attempted to investigate associations between the risk model and some common chemotherapeutics, such as Docetaxel, Gefitinib, Paclitaxel, Rapamycin, Gemcitabine, Cisplatin. The low-risk group was associated with a higher half inhibitory centration (IC50) in these chemotherapeutics, namely, the high-risk group was more sensitive to these chemotherapeutics (all p < 0.001), which implied that the model expected to be a potential predictor for chemosensitivity (Figure 7(a-f)).

### Construction and Validation of a Nomogram

**Figure 8.**
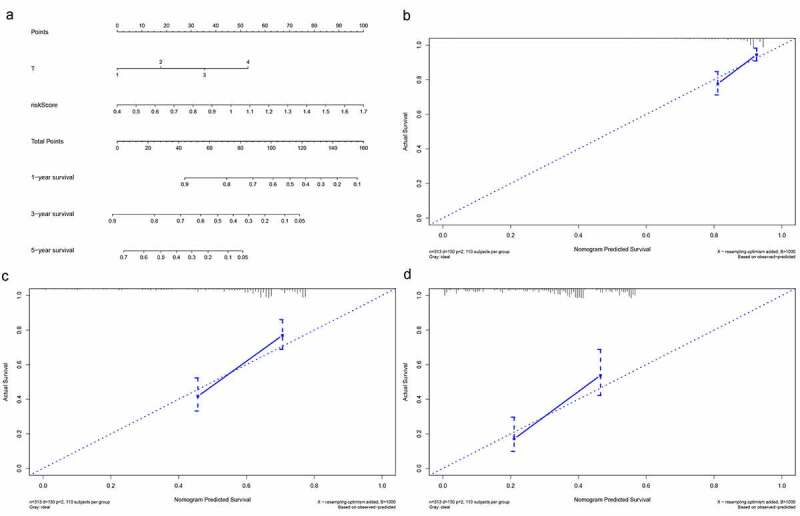
Building and validation of the nomogram predicting overall survival for lung adenocarcinoma patients. (a) The nomogram built based on the T stage and risk signature. (b, c) Calibration curves for predicting 1-, 3-, and 5-year survival of LUAD patients

As show in Figure 8, T stage and risk score, which presented as independent prognostic factors in multivariate Cox regression analysis in training set were used to develop a prognostic nomogram for predicting 1-, 3-, and 5-year survival probability of LUAD patients. The results indicated that the prediction performance of the nomogram was good (The C-index of the nomogram was 0.769 and the calibration of plots showed good agreement between the actual observations and the predictions).

## Discussion

LUAD is a common carcinoma with a very low mortality 5-year survival rate [2]. The treatment outcome is also unsatisfactory in LUAD patients because of the high mortality. In recent years, more and more researchers have found that lncRNAs played a potential role as diagnostic biomarkers, oncogenic functions and drivers of tumor suppressive in LUAD [17,18]. LncRNAs were found to participate in 33 kinds of cancers. For example, in the study of prostate cancer, Hu et al. found that lncRNAs have a regulatory role on immune response, especially the interaction to memory resting CD4 + T cells [19].

In addition, the importance of immune-related lncRNAs in tumor progression and immunotherapies has become apparent [20,21]. They can not only distinguish cancer subtypes according to specific immunological characteristics but also play a part in evaluating the development of tumor combined with the tumor immune microenvironment [22–24]. The value of immune-related lncRNA has emerged in many cancers, such as glioblastoma, ovarian cancer, bladder cancer, and human hepatocellular carcinoma [25]. Li et al. constructed a five immune-related lncRNA signature and proved that signature can as a prognostic and potential therapeutic approach for glioblastoma [26]. The novel irlncRNAs signature of bladder cancer established by Zhang et al. might be a predict method in the immunotherapy [27]. These novel fields can make a progress in the era of immunotherapy. However, the signatures of survival prediction still remain many limitations. Therefore, it is necessary to construct a suitable risk model for prognosis to enhance the survival rate of LUAD. Additionally, prognostic model that related to tumor immune infiltration in LUAD are still lacking and the analysis methods of these study were commonly based on a single immune-related lncRNA. In our study, we were attempted to establish a model as a valid predictor of LUAD by using another way – ‘immune-related lncRNA pairs,’ and investigate the role of this novel model in LUAD.

There are two sets in our study, training set and test set, the former used for constructing model, and the later use for further validating the model. Lasso regression analysis was commonly used for improving the accuracy and efficacy on risk prediction [28]. Hence, we used LASSO analysis to help us generate risk model. In our study, a six-immune-related lncRNA pairs finally participated in model construction. According to the risk model formula and the median risk scores, the samples in the training set were divided into a high-risk group and a low-risk group. In order to verify the efficiency of our model, we used a K–M survival curve and risk plot to illustrate the survival time of the two groups. The p value <0.001 in K-M analysis, which indicate that the high-risk group prone to worse prognosis as well as a higher proportion of deaths. Then the results have been confirmed in the testing set. Multivariate analysis show that risk score was independent prognostic factor either in training set or test set, which disclose that our model has a vital impact on prognosis of LUAD. In clinical work, we only need to obtain the expression of the six immune-related lncRNAs pairs, calculate the risk score based on the coefficients determine whether the patients are classified as low or high risk, then the prognosis of patients could be predicted. With advances in gene sequencing technology, this could soon become a reality.

Further, by exploring the expression situation of clinical characteristic in two groups, we also found that majority of high-risk patients was included in advanced stage of tumor. Finally, the tumor immune infiltration, ICI-biomarkers, susceptibility of chemotherapeutics in LUAD patients were probed. And the result of above analysis manifested our modeling algorithm was effective.

Tumor-infiltrating immune cells (TIICs) are often involved in the antitumor response to lung tumorigenesis [29]. Zhang et al. found that advanced stage LUAD presented lower immune scores, less memory B cells and M0 macrophages compared to early stage LUAD [30]. Here, we used seven common methods on the website to evaluate the TIICs in LUAD, and our result shows that the high-risk score was with more macrophages M0, Neutrophil and T cell CD4+ memory activated while with less B cell, T cell CD8+ compare to low-risk score. Wang et al. reported that immune scores could forecast the effect of immunotherapy and chemotherapy [31].

Immune checkpoint inhibitors targeting the PD-1/PD-L1 axis lead to durable clinical responses in many cancer patients, such as nonsmall cell lung cancer (NSCLC). Immunotherapy with anti-PD-1(PDCD1) inhibitor and anti-CTLA-4 checkpoint inhibitor have been focused on clinic. Nivolumab and Pembrolizumab, which work by target directed against PD-1 have been approved for use in treating patients with advanced NSCLC. In our study, although the expression difference of PDCD1 and CTLA-4 was not significant in two risk groups, other ICI-related biomarkers such as, CD47, CD274, and LAG3 presented the significant expression difference. CD47 can trigger evasion of tumor cells from macrophage recognition by interacting with signal regulatory protein alpha (SIRPα), has been another hot target for tumor immunotherapy [32]. Besides checkpoint blockades therapy, the efficacy of common chemotherapeutics also identified by our risk model. The results suggested that high-risk group was more sensitive to Docetaxel, Gefitinib, Paclitaxel, Rapamycin, Gemcitabine, and Cisplatin. which indicated that our model might act as a potential predictor for chemosensitivity. The immune-related lncRNA pairs in our model may become new targets for immunotherapy and lead to new therapeutic strategies. They may open up some avenues for the study in this aspect. In the end, we used a nomogram plot that related to our model to predict the LUAD prognosis. The nomogram showed that higher T stage and risk score were correlated with higher total score, which indicated worse prognoses.

Some limitations exist in this study: (1) We tried to use the datasets in the Gene Expression Omnibus (GEO) database (https://www.ncbi.nlm.nih.gov/geo/) as the test set; however, due to the sequencing method, we found the number of lncRNAs in the GEO datasets was too small to be the testing set. Therefore, we could only use an internal validation by classifying TCGA dataset into the training set and the testing set randomly and equally, which inevitably increased the bias in the study. (2) Some clinicopathological features data such as grade was lack. Hence, in future work, it is necessary for us to collect more complete data and use different datasets for further verification of our model.

## Conclusion

In conclusion, this study established a novel IRLPs model that not only predicts prognosis in patients with LUAD, but also might help in distinguishing the patients who could benefit from antitumor immunotherapy.

## Supplementary Material

Supplemental Material

## Highlights

- Six immune-related lncRNA pairs (IRLPs) were used to construct the risk assessment model.
- ROC curves suggested that our model was effective for predicting survival.
- Half inhibitory centration (IC50) of chemotherapeutics and immune cell infiltration state were significantly different between high- and low-risk group.
- Nomogram showed that higher risk score indicated worse prognosis.
- The model established by paring irlncRNAs showed a promising clinical prediction value.

## Data sharing statement

The data used to support the findings of this study are available from the TCGA open database (https://tcgadata.nci.nih.gov/tcga/; LUAD). GTF files were obtained from Ensembl (http://asia.ensembl.org).The the ImmPort database (http://www.immport.org) was used to retrieve immune-related genes (ir-genes) list.

## Financial

This work was supported by ‘333 Project’ of Jiangsu Province (Grant number: BRA2020157), ‘Six One Project,’ Research Projects of High-level Medical Personnel of Jiangsu Province (Grant number: LGY2019025), High-level Talent Selection and Training Project of The 16th Batch of ‘Six Talent Peak’ in Jiangsu Province (Grant number: WSN-245), Medical Scientific Research Foundation of Jiangsu Commission of Health (Grant number: H2018083), Jiangsu Provincial Medical Youth Talent (Jiangsu Health Scientific Education 2017 no.3), 333 High-Level Talent Training Project (Grant number: 2016, III-0719), and High-Level Medical Talents Training Project (Grant number: 2016CZBJ042).
